# Anti-SOX1 antibodies-positive paraneoplastic neurological syndromes caused by thyroid carcinoma: A case report

**DOI:** 10.1097/MD.0000000000033499

**Published:** 2023-04-21

**Authors:** Yazhi Deng, Xiaobo Zhang, Lei Wang, Xuelin Lu, Yunchun Gao, Zhenkai Wu, Zhenzhen Zhong

**Affiliations:** a Medical College of Hunan Normal University, Changsha, Hunan Province, China; b First People’s Hospital of Changde City, Changde, Hunan Province, China.

**Keywords:** anti-SOX1 antibodies, autonomic nervous system diseases, nervous system, paraneoplastic syndromes, thyroid carcinoma

## Abstract

Paraneoplastic neurological syndromes (PNSs) are a group of neurological diseases caused by distant immune effects of malignant tumors, which often occur in patients with small cell lung cancer but are not prone to occur in patients with thyroid cancer. Anti-Sry-like high mobility group box (SOX)1 antibodies (abs)-associated PNSs caused by thyroid cancer are clinically rarer.

**Patient concerns:**

A 57-year-old Chinese male patient presented with autonomic neuropathy. A thyroid biopsy revealed the diagnosis of papillary thyroid microcarcinoma. The serum anti-SOX1 abs confirmed positive.

**Diagnoses:**

A diagnosis of anti-SOX1 antibodies-positive PNS was made.

**Interventions:**

The patient received total thyroidectomy.

**Outcomes:**

After total thyroidectomy, the patient’s symptoms resolved quickly, and the serum anti-SOX1 abs test results was negative on re-examination.

**Lessons:**

Thyroid cancer can cause anti-SOX1 abs-associated PNS with only autonomic neuropathy.

## 1. Introduction

Paraneoplastic neurological syndromes (PNSs) are rare conditions associated with cancer but are not directly caused by hormones that are invaded, metastasized or secreted by tumors. The pathogenesis of PNSs are not comprehensively understood and primarily involves immune factors.^[1]^ The incidence rate of PNSs are approximately 1.0 to 1.6 per million person-years,^[2,3]^ and 1-third of the patients present with peripheral nerve damage, including somatic or autonomic nerves.^[4,5]^ The high-risk antibodies associated with PNSs (>70% associated with cancer) include anti-neuronal nuclear antibody anti-neuronal nuclear antibody-1 (ANNA-1/anti-Hu), collapsin response-mediator protein 5, anti Sry-like high-mobility group box1 (SOX1) antibodies (abs), microtubule-associated protein 1B, anti-neuronal nuclear antibody2, Purkinje cell antibody-1, amphiphysin, anti-Ma2 antibodies, and/or anti-Ma antibodies, delta/notch-like epidermal growth factor–related receptor and Kelch-like protein 11.^[6]^ Anti-SOX1 abs target Sry-like high-mobility-group developmental transcription factor superfamily proteins, which were initially found in patients with small cell lung cancer (SCLC) who did not have neurological disease.^[7]^ The presence of anti-SOX1 abs alone are an uncommon potential predictor of PNSs, usually occurring in patients with Lambert-Eaton myasthenic syndrome and paraneoplastic cerebellar degeneration.^[8]^ Up to now, only 1 case of anti-SOX1 abs-associated PNS caused by thyroid cancer has been reported, with the main symptoms being diplopia and paresthesia.^[9]^ Here, we reported a PNS case caused by thyroid cancer with autonomic neuropathy as the only manifestation.

## 2. Case

A 57-year-old male patient began to experience symptoms including frequent urination, sexual dysfunction, dry mouth and constipation approximately 2 years before admission, accompanied by syncope twice when standing, which gradually worsened. He did not have dyskinesia and could jog or even swim. He had no history of smoking, drinking, long-term exposure to poisons or heavy metals and any other disease. His supine blood pressure was 145/90 mm Hg and pulse was 70 beats per minute. His blood pressure was 80/57 mm Hg and pulse was 75 beats per minute 2 minutes after standing. Neurological physical examination revealed normal language, normal muscle tension and strength of the extremities, normal tendon reflexes of the extremities (++), no decline in cognitive function, no pathological signs, no abnormalities in deep or superficial sensation of the extremities, and negative finger-to-nose test results of both hands. The skin scratch test result was positive in both upper limbs.

**Figure 1.**
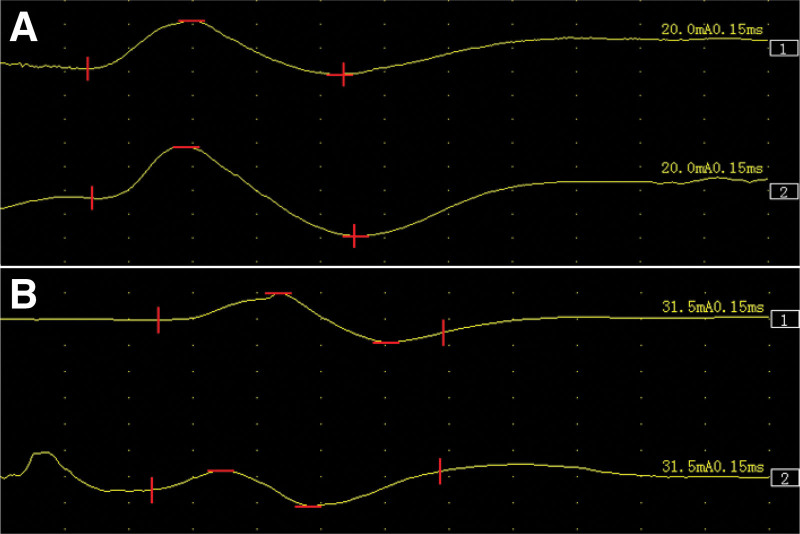
Photograph of the SSR waveform. SSR = sympathetic skin response.

**Figure 2.**
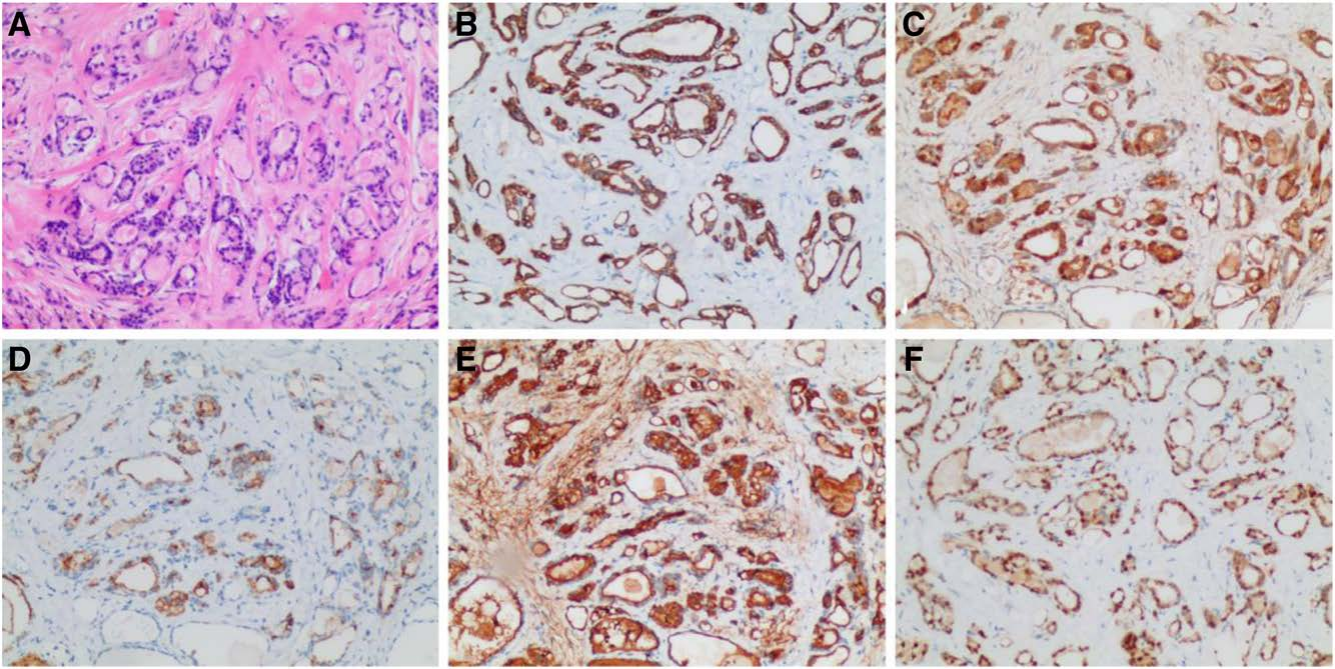
Images of H&E and IHC showing primary papillary thyroid carcinoma(200X). H&E = haemotoxylin and eosin. IHC = immunohistochemistry.

After admission, magnetic resonance imaging of the brain suggested no signs of pontine and cerebellar atrophy and no “cross sign.” In addition, electrophysiological finding shows sympathetic skin response revealed decreased amplitude of response in both upper limbs and no abnormality was observed in both lower limbs (Fig. 1), but electromyography and nerve-conduction-velocity were normal (Table 1). Urodynamic testing revealed a neurogenic bladder; Furthermore, blood routine, urine routine, stool routine, blood lipid and uric acid tests, valuation of sjogren’s syndrome antibody A, sjogren’s syndrome antibody B, thyroid-associated abs levels, erythrocyte sedimentation rate, and vitamin B1 levels were normal. Liver, kidney and thyroid function tests and colonoscopy revealed no significant abnormalities. Based on these test results, we ruled out MSA, Guillain–Barré syndrome, Sjogren’s syndrome and metabolism-related diseases. Colour Doppler ultrasonography of the thyroid gland revealed hypoechoic and heterogeneous nodules in the left lobe of the thyroid gland, (TI-RADS 4a grade) and hypoechoic nodules in the right lobe of the thyroid gland (TI-RADS 4a–4b grade), indicating the possibility of thyroid malignant tumor. Therefore, tumor markers were used for diagnosis; However, the results were negative. Needle biopsy of the thyroid gland was performed; however, no tumor cells were found. We questioned the presence of PNS but found no occult tumor after whole-body positron emission tomography–computed tomography. Analysis of patient sera by linear blotting found that anti-SOX1 abs were positive, while the Ri, ANNA-1/anti-Hu, Yo, anti-Ma2 antibodies, collapsin response-mediator protein 5, leucine-rich glioma-inactivated 1, contactin-associated protein-like2, N-methyl-D-aspartate receptor, and glutamic acid decarboxylase test results were negative. Considering false positives for anti-SOX1 abs, PNSs-related antibodies was reexamined, which yielded the same results as those of previous diagnostic evaluation. Based on the above results, we speculated that thyroid cancer was the possible etiology. About 20 days after admission, total thyroidectomy was performed, and the specimen was subjected to immunohistochemistry and haemotoxylin and eosin stain. The results showed that cytokeratin 19, galectin-3, Thyroglobulin, thyroid transcription factor 1, hector battifora and mesothelioma 1 is positive and haemotoxylin and eosin staining showed obvious ground glass nuclei (Fig. 2). According to the latest criteria for PNS, PNS-Care score for this patient is 7 (Anti-SOX1 abs and cancer) which classifies as a probable PNS.^[10]^

**Table 1:** The EMG and NCV findings of the patient.

Variable			Latency (ms)	Amplitude (µv)	NCV (m/s)
Motor fiber	Ulnar nerve	Left	2.10	10.8	46.5
		Right	1.79	10.3	48.4
	Median nerve	Left	2.74	13.4	53.6
		Right	2.78	15.5	52.4
	Tibial nerve	Left	3.78	15.5	46.1
		Right	3.50	13.7	48.1
	Common peroneal nerve	Left	3.71	6.8	46.5
		Right	3.17	5.8	46.0
Sensory fibers	Ulnar nerve	Left	1.71	19.4	55.6
		Right	1.53	20.0	65.4
	Median nerve	Left	1.96	30.6	58.5
		Right	2.00	33.8	65.0
	Superficial peroneal nerve	Left	3.10	8.8	48.4
		Right	2.38	12.9	50.4
	Sural nerve	Left	2.13	11.6	56.3
		Right	2.17	14.5	55.3

EMG = electromyography, NCV = nerve-conduction-velocity.

The symptoms of dry mouth and frequent urination were relieved 2 days following surgery, and the supine blood pressure did not change significantly. However, the blood pressure when standing increased to 110 to 120 mm Hg systolic and 60 to 65 mm Hg diastolic. Approximately 2 months after surgery, the anti-SOX1 abs test result was negative, and orthostatic hypotension and constipation continued to improve during the next 3 months. According to the follow-up, it was further confirmed that thyroid cancer can cause anti-SOX 1 abs-associated PNS with only autonomic neuropathy.

## 3. Discussion

PNSs are a rare class of neurological disorders associated with tumors, which are most commonly caused by SCLC. Autonomic neuropathy, an extremely rare PNS phenotype, often occurred in approximately 10% of patients with thymoma.^[10]^ In the present case, we describe a highly considered PNS with typical manifestations of autonomic neuropathy, the cause of which was thyroid cancer. In addition, the evaluation of anti-neuronal autoimmune antibodies revealed positive anti-SOX1 abs, whereas the evaluation of the classical diagnostic indicators including ANNA-1/anti-Hu, Yo, and Ri^[6]^ yielded normal results.

Prior research suggested that the antibodies most commonly associated with paraneoplastic autonomic neuropathy may be ANNA-1/anti-Hu, anti-CV2 or anti-ganglionic nicotinic acetylcholine receptor,^[11]^ and anti-SOX1 abs were mainly related to SCLC.^[8]^ However, in recent years, anti-SOX1 abs have been gradually found to be related to non-SCLC tumors, including breast cancer,^[4,9]^ Hodgkin lymphoma.^[12]^ To date, cases of anti-SOX1 abs-positive PNS caused by thyroid cancer has also been reported with the main symptoms being diplopia and hypesthesia.^[8,9]^ We report a case of autonomic neuropathy, wherein only the anti-SOX1 abs test result was positive among all tested onconeural antibodies.

Only a small number of patients without PNS are positive for anti-SOX1 abs or have PNS with unknown etiology, which mainly manifests as peripheral neuropathy,^[9]^ indicating that anti-SOX1 abs may mediate peripheral nerve-related immune responses. Furthermore, 66.7% of patients with positive anti-SOX1 abs do not have malignant tumors^[9]^; therefore, anti-SOX1 abs can only be used as a reference index rather than a diagnostic standard.

In general, eradicating the underlying malignancy is a crucial component of the treatment of PNS.^[11]^Moreover, removing the tumor is the most effective treatment, preventing the progression of neurological symptoms and, in some cases, improving neurological symptoms.^[10]^In a study of 200 patients with paraneoplastic encephalomyelitis associated with ANNA-1/anti-Hu, tumor treatment was an independent predictor of disease improvement or stabilization.^[13]^Autonomic dysfunction symptoms resolved after thyroid cancer resection in our study, confirming the diagnosis of PNS.

Recent studies have also found that autonomic dysfunction is associated with prostate cancer with anti-AchR antibodies.^[14]^ Similar findings were reported in our case with thyroid carcinoma. To our knowledge, this is the first case report of anti-SOX1 abs-positive autonomic PNS with a high suspicion of papillary thyroid microcarcinoma. It is unclear whether this antibody plays a pathogenic role in our patient’s PNS or represents an epiphenomenon that may be associated with the presence of thyroid cancer. This case can improve our understanding of the anti-SOX1 abs, which are helpful for early localization and prompt treatment of potential malignancies. Of course, there are shortcomings in this study, and more cases are needed to verify the relationship between thyroid carcinoma and PNS.

## Acknowledgments

The author is very grateful to the patient for agreeing to participate in this study.

## Author contributions

**Data curation:** Xiaobo Zhang, Xuelin Lu, Yunchun Gao.

**Formal analysis:** Lei Wang, Zhenkai Wu.

**Writing – original draft:** Yazhi Deng, Zhenzhen Zhong.
